# A Review of Vat Photopolymerization Technology: Materials, Applications, Challenges, and Future Trends of 3D Printing

**DOI:** 10.3390/polym13040598

**Published:** 2021-02-17

**Authors:** Marek Pagac, Jiri Hajnys, Quoc-Phu Ma, Lukas Jancar, Jan Jansa, Petr Stefek, Jakub Mesicek

**Affiliations:** Center of 3D Printing Protolab, Department of Machining, Assembly and Engineering Technology, Faculty of Mechanical Engineering, VSB-TU Ostrava, 17. listopadu 2172/15, 708 00 Ostrava-Poruba, Czech Republic; jiri.hajnys@vsb.cz (J.H.); phu.ma.quoc@vsb.cz (Q.-P.M.); lukas.jancar@vsb.cz (L.J.); jan.jansa1@vsb.cz (J.J.); petr.stefek@vsb.cz (P.S.); jakub.mesicek@vsb.cz (J.M.)

**Keywords:** vat photopolymerization, SLA, DLP, CDLP, CLIP

## Abstract

Additive manufacturing (3D printing) has significantly changed the prototyping process in terms of technology, construction, materials, and their multiphysical properties. Among the most popular 3D printing techniques is vat photopolymerization, in which ultraviolet (UV) light is deployed to form chains between molecules of liquid light-curable resin, crosslink them, and as a result, solidify the resin. In this manuscript, three photopolymerization technologies, namely, stereolithography (SLA), digital light processing (DLP), and continuous digital light processing (CDLP), are reviewed. Additionally, the after-cured mechanical properties of light-curable resin materials are listed, along with a number of case studies showing their applications in practice. The manuscript aims at providing an overview and future trend of the photopolymerization technology to inspire the readers to engage in further research in this field, especially regarding developing new materials and mathematical models for microrods and bionic structures.

## 1. Introduction

Additive manufacturing (AM) technology was first introduced in the 1980s with the aim of producing rapid and functional prototypes from various materials. Particularly, in May 1980, Dr. Hideo Kodama from the Nagoya Municipal Industrial Research Institute (NMIRI) applied for a patent that describes a method of curing a spatial model of photopolymer material by exposing it to ultraviolet (UV) light. Nevertheless, Kodama’s efforts toward finding funding for further research and development on the issued patent bore no fruit, even with support from the government. The patent was consequently abandoned and never commercialized [1,2,3,4]. Kodama is, however, credited as the first inventor of this technology.

The origin of 3D printing with commercial potential can be traced back to 1986 when Charles W. Hull filed a patent for a photopolymerization technology called stereolithography (SLA). The very first printed model was an eye-wash cup. Henceforth, SLA has been developed by 3D Systems Corporation [1,2,3].

New developments are also closely related to the development of 3D printing in medical and regenerative medicine. Dating back to 2004 in [5], a novel technique for printing 3D cell patterns was introduced. Later on, in [6], the author describes the process of manufacturing structures, namely, scaffolds, on which, cells can grow after being implemented into the body. A typical example of 3D bioprinting is artificial vessels, for which, dog bone marrow samples were used [7,8].

## 2. Vat Photopolymerization

**Figure 1:**
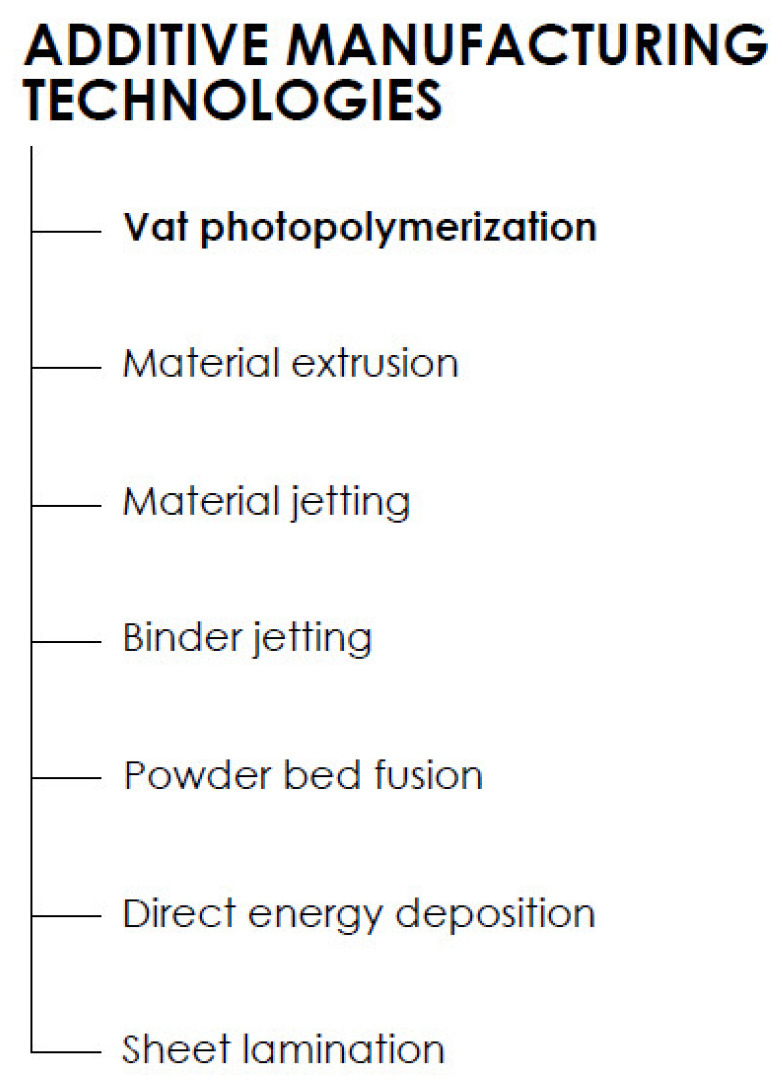
Division of the additive manufacturing (AM) technologies according to ISO/ASTM 52900: 2015 Additive Manufacturing—General Principles—Terminology.

AM technologies are classified as shown below in Figure 1. Each technology differs from another in the manner of the application process, curing principle, and the initial state of the material. Herein, attention is paid to the historically oldest AM technology, namely, vat photopolymerization.

### 2.1. 3D Printing Process

The 3D printing production process begins with a 3D model that is designed with computer-aided design (CAD) software or 3D scanned from a physical object. The 3D model is subsequently converted to the standard triangulation language (STL) format. It should be noted that since 1987, STL has been the standard and most frequently used format for preparing data for AM production. Specifically, the STL format solely describes the surface of the 3D model with a network of triangles of different sizes, depending on the required resolution. The smaller the size of the triangles, the more accurately the triangular mesh represents the desired surface, yielding a smoother surface of the to-be-printed object. The stage in which preparing the desired mesh model takes place is called preprocessing [9].

The second production stage is called processing. As a 3D printed object is fabricated layer by layer, each consecutive layer has to be supported by either the platform, the preceding layer, or extra support elements. After designing the correct and optimal orientation of the model and the supports, the STL model, including the supports, is sliced into layers with a plane parallel to the platform surface, namely, the xOy plane. Each layer is then built consecutively in the Oz direction. The layer thickness depends on the printer, AM technology, and quality requirements. The sliced model is subsequently sent to the printer. Differing from the traditional subtractive manufacturing process (machining), where the material is taken out of a workpiece, AM offers more efficient material usage.

After the printing is finished, the model is removed from the platform and other technical processing procedures are used to refine the printed object in a stage called postprocessing. In the case of photopolymerization, the as-built models are rinsed in a wash solution, most commonly, isopropyl alcohol (IPA), to get rid of the liquid layer of resin. To enhance the mechanical properties, these models are subsequently artificially cured with UV light or naturally exposed to sunlight. Other processes include support removal, grinding, sealing, gluing, polishing, painting, varnishing, coating, sterilization, inspection, and measurement. There are specific post-processing treatments for photopolymer parts. It is noteworthy that the rinsing time, temperature, and duration of the curing process play an important role in determining the mechanical properties of the finished parts.

**Figure 2:**
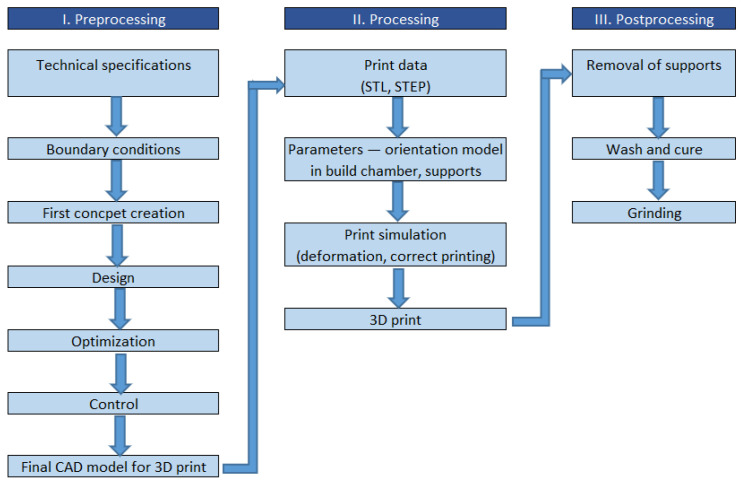
A typical production process with photopolymerization. CAD: computer-aided design, STL: standard triangulation language, STEP: standard for the exchange of product data.

A typical printing process with SLA technology is shown below in Figure 2.

### 2.2. Printing Principle of Photopolymerization

**Figure 3:**
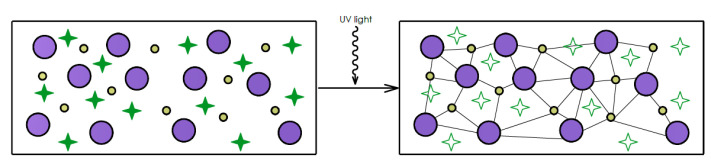
Liquid photopolymer (on the left), induced polymerization by light (small circle—monomer, large circle—oligomer, star—photoinitiator).

In the photopolymerization process, a photopolymer, which is a light-curable resin, is stored in a vat and treated with either visible or UV light. The curing light triggers and manifests the polymerization reaction, which then forms chains of polymers or crosslinks them to form a solid resin. As can be seen in Figure 3, three elements of the photopolymer mixture are monomers, oligomers, and photoinitiators. When exposed to curing light, photoinitiators release creative species that work as catalysts for the chain formation among monomers and oligomers. The chain-forming chemical-thermal process is irreversible and prototypes cannot be changed back to liquid form. Utilizing this principle, consecutive layers of resin are fabricated gradually from a sliced STL file [10,11,12,13,14].

**Figure 4:**
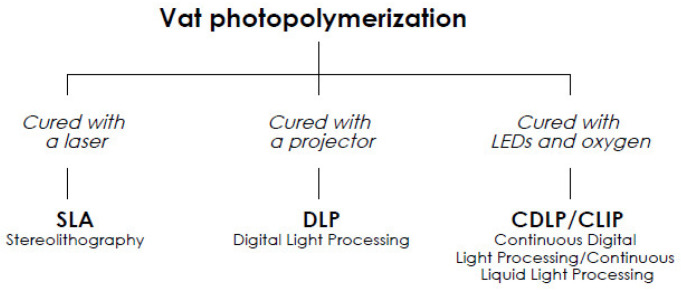
Different curing methods for vat photopolymerization. LEDs: light-emitting diodes.

Photopolymerization is classified with regard to the method of curing (Figure 4), which employ lasers (SLA), digital projection (digital light processing (DLP)), and light-emitting diodes (LEDs) and oxygen (continuous digital light processing (CDLP)/continuous liquid interface production (CLIP)) [9,15,16,17,18,19].

### 2.3. Classification

#### 2.3.1. Stereolithography

**Figure 5:**
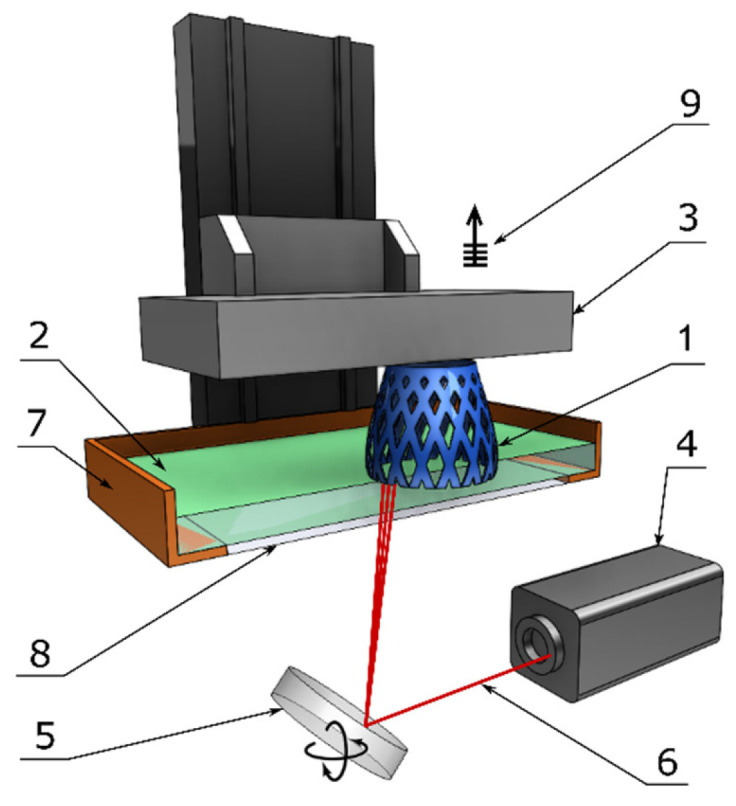
Components of a typical SLA machine: 1—printed part, 2—liquid resin, 3—building platform, 4—UV laser source, 5—XY scanning mirror, 6—laser beam, 7—resin tank, 8—window, and 9—layer-by-layer elevation.

A 3D printer with SLA technology uses a platform immersed in a transparent tank that is filled with light-curable resin (henceforth, simply resin). While the platform is immersed in the resin, the laser beam traces the construction area and cures the areas according to the sliced STL template. After a layer is formed, the platform is lowered or raised in the Oz direction, depending on whether the machine uses a top-down or bottom-up process, by a constant amount equaling the height of a layer. The curing process is repeated layer-by-layer until a 3D model is completed. The height of a layer ranges from 12 to 150 µm. In practice, 100 µm is the most chosen layer height. The print speed of standard SLA printers is in the range of 10–20 mm/h. The principle of a 3D printer with SLA technology is shown in Figure 5. The accuracy of SLA production is related to the diameter of the laser beam at the curing point, namely, the spot size. For example, for a Formlabs Form 2 SLA, the spot size is 140 µm [18,19].

#### 2.3.2. Digital Light Processing

**Figure 6:**
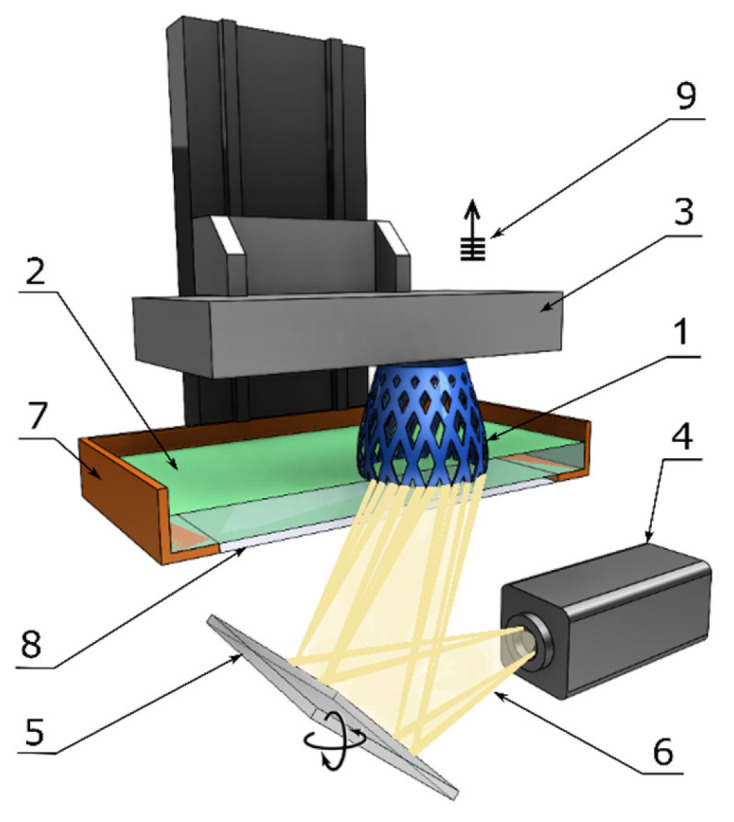
Components of a typical DLP machine: 1—printed part, 2—liquid resin, 3—building platform, 4—light source, 5—digital projector, 6—light beam, 7—resin tank, 8—window, and 9—layer-by-layer elevation.

DLP technology differs from SLA solely in the curing method. Instead of a mirror to reflect a laser source, a digital light projector is employed. In comparison with SLA technology, the DLP process is faster as each layer is entirely exposed to the curing light projected from the digital screen. Since DLP technology utilizes a digital light projector, each layer appears pixelized and the accuracy of the printed part depends greatly on the projector resolution. A typical DLP machine and its components are illustrated below in Figure 6 [18].

#### 2.3.3. Continuous Digital Light Processing/Continuous Liquid Interface Production

**Figure 7:**
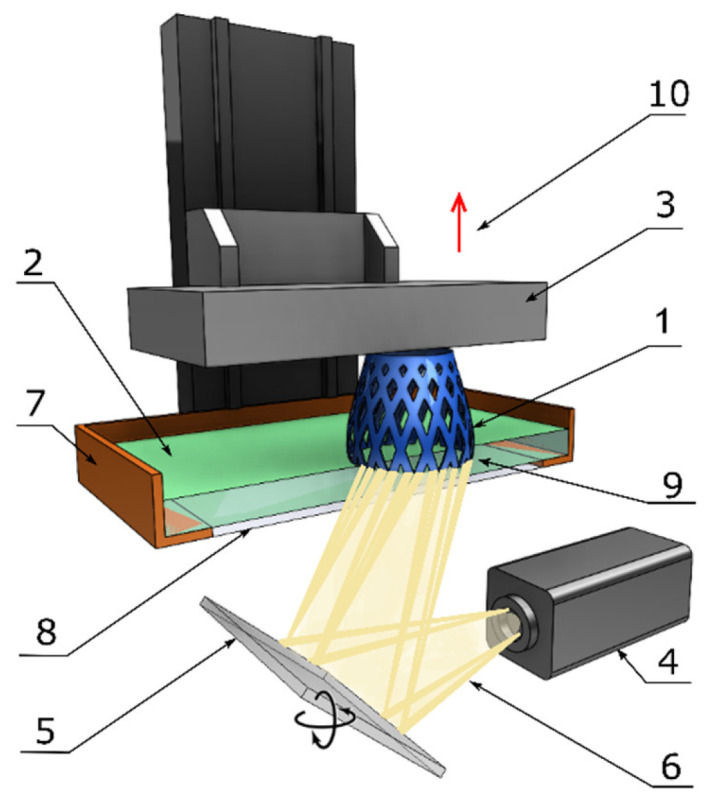
Components of a typical CDLP/CLIP machine: 1—printed part, 2—liquid resin, 3—building platform, 4—light source, 5—digital projector, 6—light beam, 7—resin tank, 8—oxygen-permeable window, 9—dead zone, and 10—continuous elevation.

CDLP/CLIP technology is an innovation based on DLP technology [16]. In particular, being different from SLA and DLP technology, CDLP/CLIP employs digital projection with LEDs and an oxygen-permeable window instead of a normal glass window. This oxygen-permeable window forms a so-called dead zone as thick as a human hair, which allows the liquid resin to flow between the interface of the printed part and the window. This uncured resin flow remarkably increases the resolution of the printed part, as well as decreasing the risk of printing failure due to the peeling force. Moreover, as opposed to the layer-by-layer method, CDLP/CLIP machines are designed with continuous movement of the build platform, thus, allowing for undisrupted prototype printing at speeds of several hundred millimeters per hour [15,16,18]. A typical CDLP/CLIP machine and its components are illustrated below in Figure 7.

Thanks to the continuous printing process, the problem regarding layer connection is eliminated and the visible staircase effect is minimized. Therefore, the fabricated parts have isotropic mechanical properties, although they may appear anisotropic [20,21]. This technology was utilized to 3D print supramolecular hydrogels, as seen in [17,18].

#### 2.3.4. Two-Photon Lithography (2PL)

2PL is a direct laser-writing (DLW) method that uses a laser beam to create 3D microstructures with a resolution below the diffraction limit. This is possible thanks to the polymerization of a photosensitive polymeric material, namely, a (photo)resist, based on the two-photon absorption (2PA) principle [22]. This technology has found its place in medical applications thanks to the ability to produce solid polymer objects, whose resolution is several nanometers. Unlike traditional 3D printing, 2PL technology can freely cure solid polymer in the resin vat, eliminating the need for material deposition in a layer-by-layer manner. Subsequently, the printed parts are treated in a suitable solvent to remove the uncured liquid resin. The smallest polymer identity in 3D printing with 2PL is an ellipsoidal 3D point, which is known as a voxel (volume pixel) [22]. It should be noted that the printing principle described above is applicable to negative photoresists, whose uncured portion dissolves in the solvent during postprocessing, while the cured portion does not.

The absorption coefficient of two photons in 2PL is defined by the equation:

(1) $$\alpha_{2}=\frac{2\hbar \omega }{I^{2}}v_{2PA}=\frac{N}{E}\sigma_{2PA},$$

where ℏ is the Dirac constant, *ω* is the laser frequency, *I* is the irradiance or power (radiation flux) per focused volume, *v* is the rate of transition of 2PA to the voxel, *N* is the density of the photoresist (number of reaction molecules in the voxel), *E* is the photon energy, *σ* is the cross-section of the 2PA (cm^4^·s/photon). This equation was taken from [23].

### 2.4. Accuracy

#### 2.4.1. Accuracy Comparison between SLA, DLP, and CDLP/CLIP

**Figure 8:**
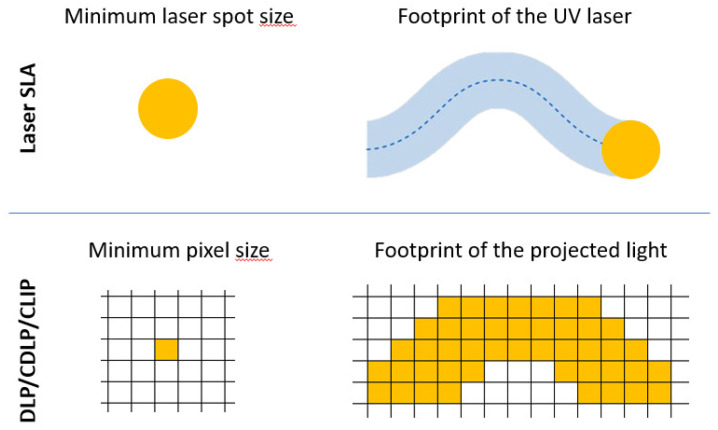
Accuracy comparison between SLA and DLP/CDLP/CLIP based on their footprint on building platform, namely, the xOy plane [24].

The curing light source and the light-projecting method play key roles in determining the accuracy of the printed parts. As for SLA, the footprint of the curing laser beam on the xOy plane is a spot and the accuracy depends on the spot diameter, whereas in DLP and CDLP/CLIP, the accuracy is given by the pixel matrix, which is defined by the resolution of the digital projector. In contrast to the flexibility that SLA offers, thanks to the free movement of the laser beam, DLP and CDLP/CLIP are limited to the network of pixels of the digital screen, which is illuminated wholly one at a time for each layer. As a result, the DLP and CDLP/CLIP deliver less accurate, Minecraft-like parts in comparison with SLA, despite its many-fold smaller individual footprint. A comparison of the production accuracies is illustrated in Figure 8 below, where a scan track in SLA is approximated with voxels in DLP and CDLP/CLIP. With regard to this fact, it can be stated that SLA technology can deliver higher accuracy than DLP and CDLP/CLIP at a cost of production time. Additionally, as aforementioned, CDLP/CLIP can fabricate parts with better accuracy and mechanical properties in comparison with DLP thanks to the continuous movement of the platform and the existence of the dead zone.

#### 2.4.2. Accuracy of 2PL

In general, there are two factors that influence the accuracy of the parts fabricated with the 2PL method, namely, the geometric error inherited from the scanning stages and the principal errors of the method itself. As 2PL printed parts are voxel-based, their accuracy primarily relies on how the voxels are spatially arranged and their resolution [25]. The 3D movement of the laser beam is controlled by a 3D piezoelectric stage, a 1D piezoelectric stage in combination with a 2D galvanometer scanner, or a 3D linear motor-driven stage with or without a galvanometer scanner. The 3D piezoelectric stage can provide a resolution of several nanometers, scanner-based systems about 100 nm, and 3D linear motor-driven stage about 400 nm [26]. These are considered geometric errors that can offset the printed parts considerably. On the other hand, the principal errors are inherited from the layer-by-layer fabricating process and from exporting the 3D model to triangular-based STL files.

## 3. Photopolymer Material Classification

After curing, the models are dimensionally stable, hard or elastic (elastomers), and can withstand very low and high temperatures. Each resin has different mechanical and chemical properties and specific uses. In general, photopolymer materials are divided into standard, structural, tough and durable, flexible and elastic, castable wax and ceramic, biocompatible, and bioink.

### 3.1. Standard Resin

Standard resin and its applications span the widest among the photopolymer materials. The most commonly used colors are grey, white, black, transparent, turquoise, and blue. Within the standard resin family, there is draft resin, which can cure 3–4 times faster than the conventional one. Thus, the draft resin is an ideal solution for quick prototyping with only one disadvantage being the height of the layer (300 µm), resulting in the stair-case effect on the surface of the model [27]. It should be noted that “green” is used to name parts that are in their as-built condition, receiving no additional post-treatments. The mechanical properties of the standard resin are summarized below in Table 1.

### 3.2. Structural Resin

A typical example of structural resin for printing universal prototypes is Grey Pro resin, which can deliver printed parts with high accuracy, slight elongation, and low creep. Thus, it is applicable for conceptual modeling, reusable functional prototypes, and precise templates for molding.

As for rigid structural resin, it can produce a combination of remarkably high rigidity and accuracy thanks to the glass reinforcement, yielding a glossy surface. In addition, this resin can be utilized to print thin walls and details, such as turbine blades, fans, connections, tooling, electronic covers, and cabinets in the automotive industry.

Among the structural resin family, there is a heat-conductive one so-called High Temp resin, which can withstand temperature up to 289 °C under a pressure of 0.45 MPa. The material is suitable for parts under light pressure and high temperatures, such as injection molding prototypes, heat-resistant fittings, hot gas, liquid piping, and electronics covers.

The mechanical properties of the three resins are summarized below in Table 2.

### 3.3. Tough and Durable Resin

Durable resin is made from polypropylene (PP) or polyethylene (PE), which has high ductility and deformation and impact resistance. It is applicable for compressible parts and assemblies with low friction and nondegrading surfaces, as well as jigs and clamps undergoing significant impacts.

Additionally, there is Tough resin (Acrylonitrile butadiene styrene (ABS)-like), which has high tensile strength and elastic modulus. The material is suitable for functional prototypes, such as jigs and clamps, which require high-stress resistance with minimal deformation.

The mechanical properties of the two resins are summarized below in Table 3.

### 3.4. Elastic and Flexible Resin

Elastomeric polyurethane (EPU) is a group of highly elastic and flexible polymers that behave similarly to injection molded polyurethane (PU) elastomers. EPU behaves elastically over a wide range of temperatures while maintaining high flexibility, i.e., it possesses low stiffness and extreme ductility. Thanks to the notably high elasticity, PU has been widely used in orthotic and prosthetic devices. A typical example is sports footwear with a microrod construction, as listed later in the Applications section.

Flexible polyurethane (FPU), on the other hand, is a semi-rigid material with remarkably high resistance to impact, shock, and repeated strains. FPU behaves similarly to injection-molded polypropylene (PP), with a mean stiffness and impact strength of 40 J·m^−1^. The FPU can be elongated by more than 280% before breaking at a stress level of 29 MPa.

For curable resins, the hardness value is in general 35 Shore D after curing; the flexible resin can reach a hardness of 80 Shore A. This material is suitable for parts withstanding deformation, bending, and compression, i.e., in stamping and packaging.

The mechanical properties of the two resins are summarized in the Table 4 below.

### 3.5. Ceramic and Castable Wax Resin

Ceramic resin is a photopolymer filled with silica. After printing and firing, the photopolymer network is burnt out, leaving the ceramic part. Similar to working with ceramic paste, the ceramic resin has high malleability and can be used for applications where the accuracy of the fired component is not critical.

Castable wax resin has been utilized to produce parts with precise details and smooth surfaces. Thus, besides being used as quick prototypes, parts printed with this material are employed as master patterns for investment casting applications. Castable wax resin, with a 20% wax fill, can facilitate reliable casting without ash and with clean melting.

The mechanical properties of the two resins are summarized in Table 5 below.

### 3.6. Biocompatible Resin

Applying photopolymerization in medical applications has drawn enormous attention from the research community. Thus, relentless efforts have been put into developing non-toxic, biocompatible resins. These materials are highly used for orthopedic, orthotic, and prosthetic purposes. The advantage of 3D printing in medicine is especially the customization, where a product can be fabricated for a specific patient. This is realized when using 3D or computed tomography (CT) scans to obtain a realistic reference for a 3D printer. Moreover, biocompatible materials are biodegradable, and thus have no negative impact on the environment.

The most commonly used biocompatible materials are dental resins, being categorized as class I (EN-ISO) 10993-1: 2009/AC: 2010, USP class VI). A typical example is the Surgical Guide Resin material from Formlabs, which is used to make sampling swabs for coronavirus disease 2019 (COVID-19) virus testing, as listed in the next section. This showcases how microrod structures and biocompatible resin can be applied to solve real-world problems. Given that the material will be in contact with human bodies, biocompatible resins must be subjected to biological evaluation and testing for cytotoxicity, genotoxicity, delayed hypersensitivity, etc. [39].

Medical resins are used for the production of a wide range of sterilizable devices and components, clamps, clips, tools for preoperative planning and surgical training, implants [40], and research and development (R&D) applications. The mechanical properties of the Medical resin are summarized below in Table 6.

### 3.7. Bioink

Other applications of photopolymerization can be observed in the case of 3D printing human tissues and organs which are functional and applied in the field of regenerative medicine and tissue engineering [44]. In the last five years, a number of start-ups, e.g., Biomodex, has commercialized its 3D tissue printing [40]. Bioink is either comprised solely of cells or most of the time mixed with hydrogel, working as a cell encapsulator after being solidified.

There are three types of bioink, namely, matrix, support, and sacrificial. Support or sacrificial bioinks are additionally utilized should the structures printed with matrix bioink be mechanically unstable. An ideal bioink has to be biodegradable, biocompatible, and non-toxic to human bodies while having sufficient rigidity and permeability to hold together cells and facilitate cell growth. As for hydrogel, it is made of hydrophilic polymers that can be consolidated with plenty of mechanisms (thermal, chemical, etc.) and photopolymerization is only one of the options [45]. Reviewing the mechanical properties of the photocrosslinking-based bioink in detail is indeed outside of the scope of this manuscript.

### 3.8. Photoresist for Two-Photon Lithography

2PL operates with positive and negative (photo)resists. As aforementioned, in the case of printing with negative (photo)resists, exposure to curing light results in the interconnection of polymer chains, making the cured portion insoluble in postprocessing solvents but not the uncured one, i.e., the structure is directly written. In contrast, the cured portion becomes soluble, where the polymer chains are broken in post-processing solvent, i.e., the reverse structure is written. Indeed, negative (photo)resists are used more often, where two typical commercially available types are SU-8 and ORMOCR [46]. Reviewing the mechanical properties of the two (photo)resists in detail is also well outside of the scope of this manuscript.

## 4. Applications

Table 7 presents the information about the applications of vat photopolymerization, along with the material, machine, technology, and notes. The applications are grouped in four scales: centimeter, millimeter, micrometer, and nanometer.

## 5. Future Trends

Trends in 3D printing are shaped by the skills and experience of users, as well as the development of printers and printing materials. Indeed, photopolymerization spans a wide variety of fields: industry and engineering, smart composites, soft robotics, flexible electronics, superhydrophobic 3D objects, medical and biomedical, prosthetics and orthotics, sports equipment, jewelry, etc. Recent studies have shown the huge potential of photopolymerization in tomographic printing, 3D nanoprinting, and 4D printing.

Current trends are moving toward multimaterial printing, which utilizes single or multinozzle systems to fabricate parts with variable strength and elasticity, living cell structures for bioprinting, and 4D printing applications. Referring to AM with such smart materials, we can begin to use the two concepts, namely, smart 3D printing and smart 4D printing.

In addition, we are seeing fundamental changes in the robotics field thanks to the development of printing materials. Progress in smart composites, soft robotics, and flexible electronics is promising to deliver improved capabilities, increased safety for human interaction, lower costs, etc. Progressive and innovative materials technologies are changing the way several mature industries operate, such as medical, industrial, automotive, and aerospace robots. Some typical examples for such an evolution are electroactive polymers or artificial muscles, electroadhesion gripping, and electrolaminates [74].

Furthermore, photopolymerization has found its place in fabricating superhydrophobic objects for water-related applications, e.g., module spacers, novel filtrations, desalination membranes, adsorbents, and water remediation. The promising potential, challenges, and prospects of AM and the materials for water resource and treatment-related applications were all discussed in [74].

In the field of biocompatible materials, attention is paid to developing monomer-free resins to meet health and safety standards. Deploying these materials, dentists and orthodontists can create long-lasting and highly performing dental and other body prostheses without fear of toxicity or side effects. Other developments focus on fiber-reinforced and two-photon resins for 3D printing at the nano level [29,33].

**Figure 9:**
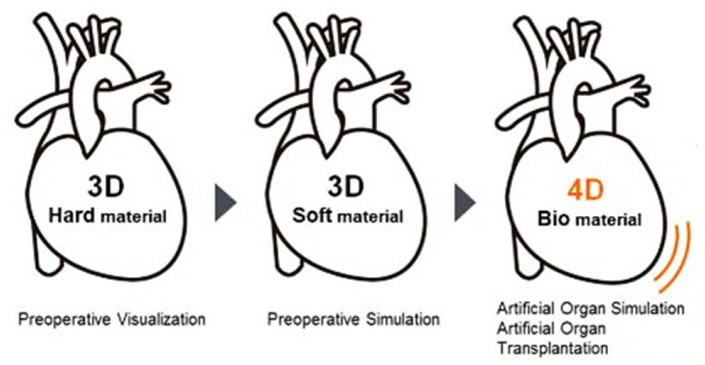
Evolution of 3D bioprinting.

**Figure 10:**
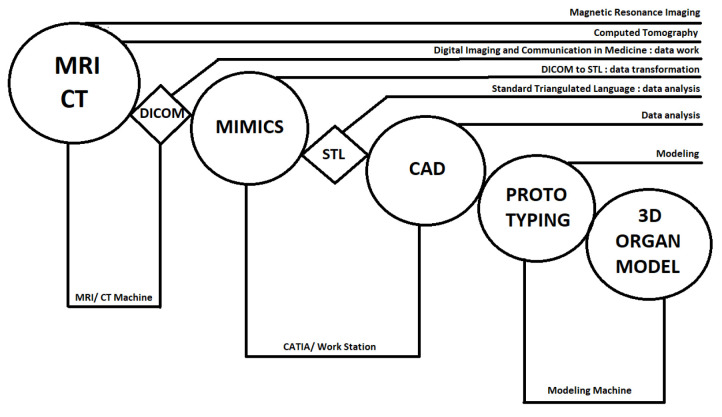
Technological process of a 3D-printing production applied in regenerative medicine [80].

Interest in 3D bioprinting organs is growing and aims to alleviate the shortage of donated organs and eliminate illegal organ trading on the black market. Another advantage is the ability to test new drugs on printed tissues (kidneys and liver) without burdening and exposing the patient’s organs to side effects. Taking another step further, 4D printing produces 3D biological parts whose shapes respond following time-dependent functions. Indeed, time is the additional 1D and the shape change is activated with stimuli from the environment, e.g., water, temperature, or light [75]. Summarizing from [8,75,76,77,78,79], the evolution of 3D bioprinting, Figure 9, and its process, Figure 10, are shown.

## 6. Conclusions

Photopolymerization in 3D printing has been used for applications in fabricating rapid and functional prototypes, customized products, and serial production. Over more than 30 years of development on photopolymerization has made great progress on improving the production methods (SLA, DLP, CDLP/CLIP), production speed, and quality of 3D printed products for industrial and research applications. Material-wise, efforts will further be paid to high-performance resins, especially biocompatible resins, and bioink.

This article serves as a review of vat photopolymerization printing technology and acquaints a wide audience of readers across various disciplines with the possibilities of their use, as well as the advantages and disadvantages of this technology.

In this review, we aimed to summarize all the various types of this technology and the large range of materials used, as well as providing specific applications of this technology in practice, especially in the field of biomedicine and customization. We think that this review is a good follow-up to the already published review of 3D printing, and it is a great addition to this article such that together they form a comprehensive overview of these latest trends in AM.

The future of photopolymerization lies in two main categories: material development, especially in medical applications, and applying modern designing methods. Indeed, there is huge potential for material development and the mathematical modeling of 4D printing using multiphysical properties, i.e., variable surface hardness, shape memory, etc. As for the construction methods, the future lies in fabricating topologically optimized, bionic, and microrod structures.

Applications of 3D printing and, in particular, photopolymerization has spanned across all industries, e.g., in robotics, medicine, dental, engineering, automotive, aerospace, and water resources and related treatments. To conclude, it can be stated that the future of 3D printing is solely limited by the creativity and technical thinking of designers.

## Figures and Tables

**Table 1:** Mechanical properties of standard resin [28,29].

Properties	Standard				
	Standard (Grayscale, Clear, Colors)		Draft Resin		
	Green ^1^	Postcured ^2^	Green ^3^	Postcured at Room Temperature ^4^	Postcured at 60 °C ^5^
Tensile Properties					
Ultimate Tensile Strength	38 MPa	65 MPa	23 MPa	28 MPa	36 MPa
Tensile Modulus	1.6 GPa	2.8 GPa	0.9 GPa	1.3 GPa	1.6 GPa
Elongation at Failure	12%	6.2%	17%	10%	7%
Flexural Properties					
Flexural Modulus	1.25 GPa	2.2 GPa	0.6 GPa	0.9 GPa	1.5 GPa
Flexural Stress at Break	-	-	-	-	-
Impact Properties					
Notched Izod	16 J/m	25 J/m	35 J/m	35 J/m	21 J/m
Temperature Properties					
Heat Deflection Temp. @ 264 psi	42.7 °C	58.4 °C	43.3 °C	44.3 °C	50.1 °C
Heat Deflection Temp. @ 66 psi	49.7 °C	73.1 °C	50.6 °C	50.7 °C	63.4 °C

^1^ Data was obtained from green parts, printed using a Form 2, 100 μm, clear settings, without additional treatments. ^2^ Data was obtained from parts printed using a Form 2, 100 μm, clear settings, and postcured with 1.25 mW/cm^2^ of 405 nm LED light for 60 min at 60 °C. ^3^ Data was obtained from green parts, printed using a Form 2, 300 µm, draft resin settings, washed for 5 min in Form Wash, and air-dried without a postcure. ^4^ Data was obtained from parts printed using a Form 2, 300 µm, draft resin settings, and postcured with Form Cure at room temperature for 5 min. ^5^ Data was obtained from parts printed using a Form 2, 300 µm, draft resin settings, and postcured with Form Cure at 60 °C for 5 min.

**Table 2:** Mechanical properties of structural resin [30,31,32].

Properties *	Grey Pro		Rigid		High Temp		
	Green ^1^	Postcured ^2^	Green ^3^	Postcured ^4^	Green ^5^	Post-Cured ^6^	Post-Cured + Thermally Postcured ^7^
Tensile Properties							
Ultimate Tensile Strength	35 MPa	61 MPa	40 MPa	75 MPa	20.9 MPa	58.3 MPa	51.1 MPa
Young’s Modulus	-	-	-	-	-	-	-
Tensile Modulus	1.4 GPa	2.6 GPa	2.2 GPa	4.1 GPa	0.75 GPa	2.75 GPa	2.9 GPa
Elongation	32.5%	13%	13.3%	5.6%	-	-	-
Elongation at Break	-	-	-	-	14%	3.3%	2.4%
Flexural Properties							
Flexural Modulus	0.94 GPa	2.2 GPa	1.37 GPa	3.7 GPa	0.69 GPa	2.62 GPa	2.62 GPa
Flexural Stress at 5% Strain	39 MPa	86 MPa	49 MPa	121 MPa	-	-	-
Flexural Strength at Break	-	-	-	-	24.1 MPa	94.5 MPa	93.8 MPa
Impact Properties							
Notched IZOD	-	18.7 J/m	-	18.8 J/m	32.8 J/m	18.2 J/m	24.2 J/m
Temperature Properties							
Head Deflection Temp. @ 1.8 MPa	-	62.4 °C	-	74 °C	43.6 °C	99.2 °C	101 °C
Heat Deflection Temp. @ 0.45 MPa	-	77.5 °C	-	88 °C	49.3 °C	142 °C	238 °C
Thermal Expansion (−30 to 30 °C)	-	78.5 µm/m/°C	-	53 µm/m/°C	-	-	-
Thermal Expansion (0 to 150 °C)	-	-	-	-	118.1 µm/m/°C	79.6 µm/m/°C	74 µm/m/°C

* Material properties can vary with the part geometry, print orientation, print settings, and temperature. ^1^ Data was obtained from green parts, printed using a Form 2, 100 μm, Grey Pro settings, without additional treatments. ^2^ Data was obtained from parts printed using a Form 2, 100 μm, Grey Pro settings, and postcured with Form Cure for 120 min at 80 °C. ^3^ Data was obtained from green parts, printed using Form 2, 100 μm, rigid settings, without additional treatments. ^4^ Data was obtained from parts printed using a Form 2, 100 μm, rigid settings, and postcured with a form cure for 120 min at 80 °C. ^5^ Data was obtained from green parts, printed using a Form 2, 100 μm, High Temp settings, washed for 5 min in Form Wash, and air-dried without a post-cure. ^6^ Data was obtained from parts printed using a Form 2, 100 μm, High Temp settings, and postcured with Form Cure at 80 °C for 120 min. ^7^ Data was obtained from parts printed using a Form 2, 100 μm, High Temp settings, and postcured with Form Cure at 60 °C for 60 min plus an additional thermal cure in a lab oven at 160 °C for 90 min.

**Table 3:** Mechanical properties of durable and tough resin [33,34].

Properties	Durable		Tough	
	Green ^1^	Postcured ^2^	Green ^1^	Postcured ^3^
Tensile Properties				
Ultimate Tensile Strength	13 MPa	28 MPa	26 MPa	33 MPa
Young’s Modulus	-	-	-	-
Tensile Modulus	0.24 GPa	1 GPa	0.94 GPa	1.5 GPa
Elongation at Failure	75%	55%	69%	51%
Flexural Properties				
Flexural Modulus	0.04 MPa	0.66 GPa	0.44 GPa	1.4 GPa
Flexural Strength	-	-	15 MPa	39 MPa
Flexural Stress at 5% Strain	1.0 MPa	24 MPa	-	-
Impact Properties				
Notched IZOD	127 J/m	114 J/m	72 J/m	67 J/m
Unnotched IZOD	-	-	902 J/m	1387 J/m
Thermal Properties				
Heat Deflection Temp. @ 1.8 MPa	-	-	34 °C	45 °C
Heat Deflection Temp. @ 0.45 MPa	<30 °C	41 °C	42 °C	52 °C
Thermal Expansion	124 µm/m/°C	106 µm/m/°C	114 µm/m/°C	97 µm/m/°C

^1^ Data was obtained from green parts, printed using a Form 2, 100 μm, clear settings, without additional treatments. ^2^ Data was obtained from parts printed using a Form 2, 100 µm, and postcured with Form Cure for 120 min at 60 °C. ^3^ Data was obtained from parts printed using a Form 2, 100 µm, and postcured with Form Cure for 60 min at 70 °C.

**Table 4:** Mechanical properties of elastic and flexible resins [35,36].

Properties	Elastic			Flexible	
	Green ^1^	Postcured ^2^		Green ^1^	Postcured ^3^
Tensile Properties					
Ultimate Tensile Strength	1.61 MPa ^4^		3.23 MPa ^4^	3.3–3.4 MPa ^4^	-
Elongation at Failure	100%		100%	60%	75–85%
Stress at 50% Elongation	0.92 MPa		0.94 MPa	-	-
Stress at 100% Elongation	1.54 MPa		1.59 MPa	-	-
Other Properties					
Hardness Shore	40 A		50 A	70–75 A	80–85 A
Vicat Softening Point	-		-	231 °C	230 °C

^1^ Data was obtained from green parts, printed using a Form 2, 100 μm, clear settings, without additional treatments. ^2^ Data was obtained from parts printed using a Form 2, 100 µm, elastic settings, washed in Form Wash for 20 min, and postcured with Form Cure at 60 °C for 20 min. ^3^ Data was obtained from parts printed using a Form 2, 100 µm, flexible settings and postcured with 80.5 mW/cm^2^ of 365 nm fluorescent light for 60 min. ^4^ Tensile testing was performed after 3+ hours at 23 °C using a Die C dumbbell and a 20 in/min crosshead speed.

**Table 5:** Mechanical properties of ceramic and castable wax resins [37,38].

Properties	Ceramic			Castable Wax
	Green ^1^	Fired ^2^		
Tensile Properties				
Ultimate Tensile Strength	5.1 MPa		-	11.6 MPa
Young’s Modulus	-		-	220 MPa
Tensile Modulus	1.03 GPa		50 GPa	-
Elongation at Failure	1.4%		-	13%
Flexural Properties				
Flexural Modulus	994.6 MPa		-	-
Flexural Stress at Break	10.27 MPa		33.5 MPa	-
Impact Properties				
Notched IZOD	18.42 J/m		-	-
Temperature Properties				
Heat Deflection Temp. @ 264 psi	74.7 °C		-	-
Heat Deflection Temp. @ 66 psi	>290 °C		-	-
Other Properties				
Cold Crushing Strength	-		72.2 MPa	-
Shear Modulus	-		21.9 GPa	-
Poisson’s Ratio	-		0.140	-
Density	-		1.9 g/cm^3^	-

^1^ Data was obtained from green parts, printed using a Form 2, 100 μm, Clear settings, without additional treatments. ^2^ Data was obtained from fired parts, printed using a Form 2, 100 μm, Ceramic settings, and washed and dried without an additional postcure. The parts were printed with a preapplied scale factor and fired using a 30 h schedule to a maximum firing temperature of 1275 °C, as laid out in the Formlabs usage guide.

**Table 6:** Mechanical properties of dental resins [41,42,43].

Properties	Medical		
	Dental LT Clear	Dental SG	Surgical Guide Resin
Ultimate Tensile Strength	-	>50 MPa	73 MPa
Young’s Modulus	-	-	2.9 GPa
Elongation at Failure	-	-	12.3%
Flexural Modulus	>1300 MPa	>1500 MPa	2500 MPa
Flexural Stress at Break	-	-	103 MPa
Hardness Shore	80–90 D	>80 D	67 D

**Table 7:** Photopolymerization applications.

Scale	Application	Material	Machine	Technology	Note	Ref.
cm	Football helmet liner	Polyurethane elastomer (EPU40)	Carbon M2	CDLP	Helmet liner constructed with 140,000 interconnected struts for impact attenuating purpose.	[47]
cm	Shoe soles (orthotics/aesthetics)	Polyurethane elastomer (EPU40)	Carbon M2	-/Digital Light Synthesis (DLS)	Customized shoe soles for orthotics/shoe soles from lattice structures for optimal usage and aesthetics.	[48,49]
mm	Artificial ears	-	Roland ARM-10	Stereolithography	Ear reconstruction based on 3D scans from patients.	[50]
mm	Hearing aids	Light-cured acrylic resin	-	-	Customized hearing aid shells from 3D scanned ear canals.	[51]
mm	Sampling swabs	Surgical Guide Resin	FormLabs Form 3/Carbon M2	CDLP	Sampling swabs with plastic heads for collecting efficiency and comfort.	[52]
mm	Jewelry	Castable wax resin	Form 3, Form 3L	Low force stereolithography (LFS)	Detailed jewelry designs are 3D-printed for investment casting.	[53]
mm	Scaffolds for cells	Accura SI10	Scanlabs	2PL	Scaffolds work as living cell encapsulators.	[54]
µm	Microneedles	Photopolymer	Nanoscribe GT	2PL	Mosquito-liked microneedles.	[55]
µm	Probes for atomic force microcopy	Resist (IP-Dip)	Photonic Professional GT	2PL	Printing tips can be carbonized by utilizing a pyrolysis process.	[56]
mm/µm	Flexible electronics	Silicon elastomers with nanosilica/ Conductive and dielectric elastomeric materials	-	-	Soft sensors, actuators, and robots to improve human–machine interactions. Strain sensors embedded into a glove shape.	[57,58,59,60,61]
mm/µm	Smart composites	Viscoelastic inks	-	-	Porous, elastomeric architectures with a programmable Poisson ratio and mechanical properties utilizing the ordered arrangement of sub-millimeter struts.	[57,58]
mm/µm	Superhydrophobic objects	Inks	-	DLP	Objects with special surface structures that are utilized for self-cleaning, drag reduction, increased buoyancy, and air-conditioning applications.	[62,63,64]
mm/µm	Living tissue structures	Bioinks	-	-	Small-scale, simplified liver, kidney, or lung tissue, mimicking their natural counterparts.	[65,66]
mm/µm	4D-printed actuators	Liquid crystalline polymers (LCPs)	-	-	Stimuli-responsive liquid crystalline elastomeric structures. The printing process prescribes a reversible shape-morphing behavior, offering a new paradigm for active polymer system preparation.	[67,68]
µm	Tomographic 3D printing (fabrication of advanced and functional constructs)	Photopolymer	-	DLP	Object is simultaneously solidified by irradiating a liquid photopolymer volume from multiple angles with dynamic light patterns.	[69,70]
nm	3D nanoprinting	Photopolymer	-	Electron/X-ray lithography	Multiphoton polymer cross-linking evolves as the core process behind high-resolution additive microfabrication with soft materials for implantable/wearable electronics, tissue engineering, microrobotics, biosensing, drug delivery, etc.	[71,72,73]
